# Supplementary material for: Efficiency of the Brain Network Is Associated with the Mental Workload with Developed Mental Schema
Source: Brain Sci. 2023 Feb 21;13(3):373. doi: 10.3390/brainsci13030373 (PMC10046556; doi:10.3390/brainsci13030373)
Supplement: Supplementary file 1 [file brainsci-13-00373-s001.zip › brainsci-2119474-Supplementary Materials.pdf]

brain sciences

Supplemental Materials for "Efficiency of the brain network is associated to the mental workload with developed mental schema (brainsci-2119474)".

subjective workload ratings. The results of subjective task difficulty assessment data for L0, L1, L2, and L3 across subjects are included in Supplemental Figure S1.

To simulate the real task scenarios, we introduced different wind parameters to induce the difference in task difficulties. In the first three scenarios (L0-L2), we controlled the wind direction as a fixed factor, i.e., the increase in wind strength made the task more difficult, which was quite intuitive. In the fourth scenario (L3), we introduced another variable, the change of wind direction, which was expected to make the task more difficult than the L3 scenario. But the actual result was not as expected. As shown in Figure S1.A, the subjective scale of the perceived difficulty revealed no significant difference between L2 and L3 scenarios, while there was a significant difference in the task difficulty of the first three scenarios (L0-L2). Taking a closer look at the distribution of scales as shown in the Figure S1B (see below), less than half found L3 more difficult than the L2 scenario, indicating that the L3 scenario failed to induce the task to become more difficult.

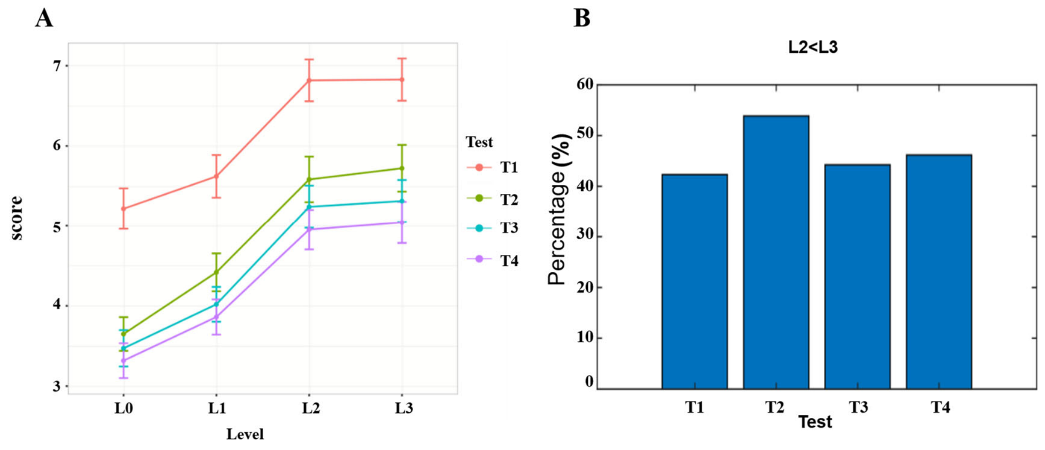

Figure S1. (A) Average subjective workload ratings for scenario types and test orders (Fig.4A of our previous publication). (B) comparisons of subjective workload ratings between L2 and L3 scenarios.
